# Commissioning, clinical implementation, and performance of the Mobetron 2000 for intraoperative radiation therapy

**DOI:** 10.1002/acm2.12027

**Published:** 2017-01-19

**Authors:** Landon S. Wootton, Juergen Meyer, Edward Kim, Mark Phillips

**Affiliations:** ^1^ Department of Radiation Oncology University of Washington School of Medicine Seattle WA USA

**Keywords:** Commissioning, intraoperative, IORT, Mobetron, mobile accelerator

## Abstract

The Mobetron is a mobile electron accelerator designed to deliver therapeutic radiation dose intraoperatively while diseased tissue is exposed. Experience with the Mobetron 1000 has been reported extensively. However, since the time of those publications a new model, the Mobetron 2000, has become commercially available. Experience commissioning this new model and 3 years of data from historical use are reported here. Descriptions of differences between the models are emphasized, both in physical form and in dosimetric characteristics. Results from commissioning measurements including output factors, air gap factors, percent depth doses (PDDs), and 2D dose profiles are reported. Output factors are found to have changed considerably in the new model, with factors as high as 1.7 being measured. An example lookup table of appropriate accessory/energy combinations for a given target dimension is presented, and the method used to generate it described. Results from 3 years of daily QA measurements are outlined. Finally, practical considerations garnered from 3 years of use are presented.

## 1 Introduction

Intraoperative radiation therapy (IORT) aims to maximize the therapeutic ratio, which represents the balance between tumor control and normal tissue toxicity. At the time of tumor resection, it may be possible to provide a direct path between an accelerator and the tumor bed since the overlying tissues are moved out of the way. When the resection leaves behind a thin region of unresected tumor cells (either microscopic or macroscopic), the use of intraoperative high‐dose‐rate brachytherapy (IORT‐HDR), low energy photons, or megavoltage electrons can provide a therapeutic dose to the tumor bed while minimizing damage to distal tissues. Accordingly, IORT has found several clinical applications using a number of different delivery devices for sites such as sarcomas,^1^, ^2^, ^3^ breast,^4^, ^5^ recurrent head and neck cancer,^6^ pancreatic cancer,^7^ locally advanced and recurrent GYN tumors,^8^ rectal cancers,^9^, ^10^ and genitourinary cancers.^11^

Delivery of IORT is most easily performed in the operating theater (OR) within a sterile environment to avoid transferring the patient from the operating theater to a linear accelerator in a radiation oncology department. Several accelerators capable of being placed in an operating room have been marketed:

- Intrabeam, Carl Zeiss Meditec, Jena, Germany (low energy photons);^12^
- LIAC, Sordina IORT Technologies, Vicenza, Italy (4*, 6, 8, 10, 12* MeV electrons, *not available on all models);^13^
- Mobetron, IntraOp Medical Corporation, Sunnyvale, CA (6, 9, 12 MeV electrons); and
- NOVAC, Sordina IORT Technologies, Vicenza, Italy (4, 6, 8, 10 MeV electrons).^13^

To some extent, the commissioning of such a system is very similar to the commissioning of a normal therapeutic megavoltage electron linear accelerator or superficial unit. However, there are a number of important differences as well.^14^ These differences include the lack of isocentricity and the use of dedicated applicators that are markedly different than conventional electron applicators.

This report details the implementation of an IORT program at our institution using the Mobetron, manufactured by Intraop Medical Corporation. This device presents a number of challenges for the medical physicist. These include the fact that many are used in unshielded rooms. The design of applicators for oblique treatments makes commissioning and dose planning challenging, particularly given the lack of a computer‐aided planning system. We describe our approaches to these issues over the past 3 years.

Experience commissioning a previous model of the Mobetron, the Mobetron 1000, has been reported previously.^15^ However, in the time since that publication a new model has become commercially available, the Mobetron 2000. In light of this, we focus on (a) characteristics that have changed in the new model (such as output factors), (b) supplemental measurements not included in previous publications, and (c) historical use data and experience at our institution. We devote minimal attention to items extensively covered elsewhere such as radiation protection shielding and TG‐51 measurements. Overall, our goal is to provide the community with a broad set of beam parameters for comparison and to perhaps provide some guidance to help others develop their own programs using this modality.

## 2 Methods

We begin by describing the Mobetron and its accessories, focusing on changes in the most current model. We then present our experience, dividing it into three categories: radiation protection, commissioning and dosimetry, and clinical procedures.

### 2.A Mobetron description

The Mobetron is a dedicated mobile electron linear accelerator designed to deliver radiation during surgery. It uses X‐band frequencies for electron acceleration in order to achieve smaller dimensions and a reduced weight more amenable to use in an operating suite. The Mobetron 2000 is an improvement in this regard, with a total weight of approximately 3000 lbs. compared to roughly 4000 lbs. for the prior model. It is also a few inches shorter to accommodate lower ceiling heights. The Mobetron 2000 produces electrons with nominal energies of 6, 9, and 12 MeV at dose rates up to approximately 10 Gy per minute. The 4 MeV setting available in the previous model was eliminated to reduce QA load, instead bolus is used to achieve a similar effect. It is designed without a bending magnet to reduce leakage radiation so that it can be used in operating rooms with minimal shielding (e.g., designed for diagnostic imaging) or no shielding. It also incorporates a beam stop which attenuates approximately ±20° of the small quantity of bremstrahlung scatter created.

The accelerator and beam stop are mounted on a gantry with a limited rotational range of ± 45°. The accelerator itself can also tilt forward and backward (−10°/+30°), and translate in the vertical (30 cm), lateral (10 cm), and longitudinal (10 cm) directions.

There are 45 cylindrical anodized aluminum applicators, or cones, each 32 cm long. They range in diameter from 3.0 to 10.0 cm in 0.5 cm increments. For each diameter, there are three applicators with distal bevel angles of 0°, 15°, and 30°. The need for the beveled end is a reflection of the rotational capabilities of the Mobetron and the limitations on access to the tumor bed. The beveled applicators are for cases in which the tumor bed lies at some angle to the horizontal that is not within the Mobetron's range of motion and/or because of anatomical constraints with respect to the resection cavity. The beveled end of the applicator may be placed flush with the tumor bed while the applicator axis is at some angle to the normal axis. Two plastic boluses (0.5 or 1.0 cm thick) are available for each applicator that fit into the distal end to provide buildup and to reduce dose to critical structures that might lie beneath the tumor bed.

**Figure 1:**
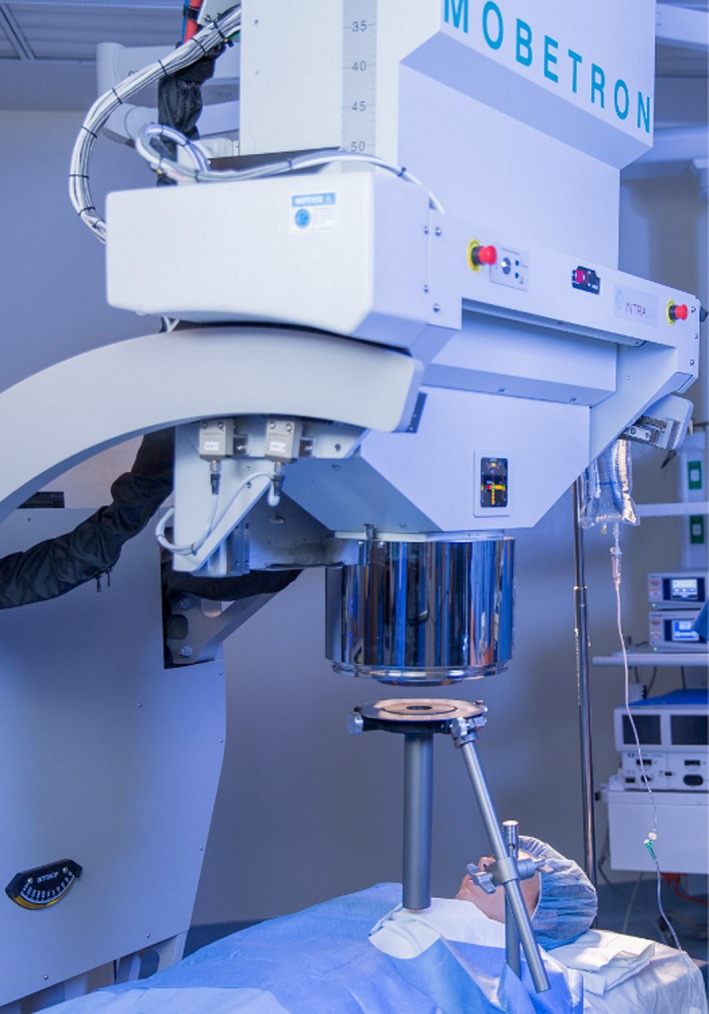
Mobetron soft docking illustration. The Mobetron head aligns with but does not contact the applicator. Alignment is facilitated by lasers in the Mobetron head which are reflected by mirrors in the applicator collar to provide feedback on positioning. The applicator and collar are clamped to the surgical table. Photo courtesy of IntraOp Medical Corporation.

The Mobetron uses a soft docking system in which the accelerator is physically decoupled from the applicator which touches the patient (Fig. 1). The Mobetron 2000 includes an improved docking algorithm intended to reduce the time required to achieve docking. A clamp is secured to the operating table and several pivoting arms hold a collar directly above the treatment site. The collar serves two purposes. The first is to securely hold the applicator itself — one end of which is fixed to the collar while the other rests on the tissue to be treated. Secondly, the other side of the collar (facing the linac) holds an annular mirror. A laser‐detector scheme in the linac head is used to provide feedback regarding the absolute alignment (translation and rotation) of the linac and applicator. There is approximately a 4 cm gap between the collar and the end of the linac head.

For commissioning purposes, there is a spacer that attaches applicators directly to the head. This spacer places the applicator the correct distance from the source (maintaining the 4 cm air gap distance), and it obviates the need for a clamp and alignment procedure at each applicator change. A separate applicator with attached phantom is used for daily quality assurance measurements. It is intended to measure values of the depths of nominal d_max_ and R_50_ for each energy for output and energy verifications prior to treatment.

Other differences between the 2000 and 1000 include a solid state modulator included in the treatment unit. This eliminates the stand‐alone modulator used by the 1000 which facilitates easier transportation and setup by reducing external cabling. The 2000 is powered by single‐phase input, to minimize or eliminate the need for electrical modification of the OR suite. The treatment module itself rests on a powered jack for faster, easier transport. The control system is housed separately in a mobile cabinet which is placed outside the OR suite.

### 2.B Radiation protection

Several publications^16^, ^17^ have documented the three‐dimensional dose distributions resulting from leakage, scatter, and transmission through the beamstopper. Krechetov et al.^18^ report such measurements for the current model of the Mobetron. Since a thorough mapping of the dose in the OR and environs requires extensive beam‐on time which was not possible in our situation given possible exposure to operators, ancillary personnel, and patients/visitors, we performed a selective set of measurements and compared these with expected values from the aforementioned publications. The feasibility of using the Mobetron in a given OR and the limits on the number of allowable monitor units were based on these publications and our measurements.

### 2.C Dosimetric measurements

All dosimetric measurements were performed in a shielded linac vault in our department to permit the extensive beam‐on time required for a comprehensive dosimetry characterization. Unless otherwise noted, all measurement were acquired in OmniPro Accept (IBA Dosimetry GmbH, Schwarzenbruck, Germany) with an IBA Electron Field Detector 3G and Reference Dosimetry Diode 3G in continuous ratio acquisition mode. Measurements were made in water with an IBA Blue Phantom and CU500E controller. Percent depth dose (PDD) and profile data were processed in OmniPro Accept.

#### 2.C.1 Output factors and air gap measurements

Output factors were measured for each applicator diameter, bevel, and energy combination. The rationale for the geometry of the measurements was described in the AAPM Task Group 48 report.^19^ The applicators were positioned so that the beveled end was flush with the water surface. To position the diode, first a vertical depth–dose profile was measured, centered under the beveled end of the applicator. Then, an in‐plane profile was measured at the depth of d_max_ determined from the depth–dose profile. The diode was positioned at the location of d_max_ of the in‐plane profile and centered in the cross‐plane for the output factor measurement. This was done to account for the fact that the beveled applicator axes were not normal to the water. Output factor measurements were normalized to the measurement for the 10‐cm 0° applicator.

Air gap measurements were performed for each energy with 4, 7, and 10 cm diameter 0° bevel cones. Measurements were performed in water with an electron diode at the depth of d_max_. The applicator was initially placed flush with the water surface and subsequently retracted to measure with 0, 1, and 2 cm gaps. Air gap factors were produced by normalizing measurements to the 0 cm gap.

#### 2.C.2 Percent depth dose and lateral profiles

PDDs were acquired for every applicator, bevel and energy combination, normal to the water surface (i.e., not along the applicator axis for beveled applicators), and centered under the beveled end of the applicator. In‐plane and cross‐plane profiles were also acquired at a variety of depths for each combination of parameters.

#### 2.C.3 2‐D dose distributions and accessory lookup table

An EBT 3 radiochromic film (Ashland Inc., Covington, KY, USA) was used to measure the relative two dimensional in‐plane dose for a subset of all possible measurements. The film was placed between two 5‐cm thick slabs of solid water, held together tightly with bar clamps. This minimized air gaps in the vicinity of the film. Care was taken to ensure the edge of the film was flush with the solid water for PDD measurements. The cone was lowered until it was level with and in contact with the solid water. Profiles were taken for each bevel angle and energy combination using 4, 7, and 10 cm diameter cones. All films were scanned on an Epson V750 flatbed scanner (Seiko Epson Corp., Tokyo, Japan) and converted to relative dose using a triple‐channel dosimetry algorithm.^20^, ^21^, ^22^ Each planar profile was normalized to d_max_ to produce isodose distributions.

**Figure 2:**
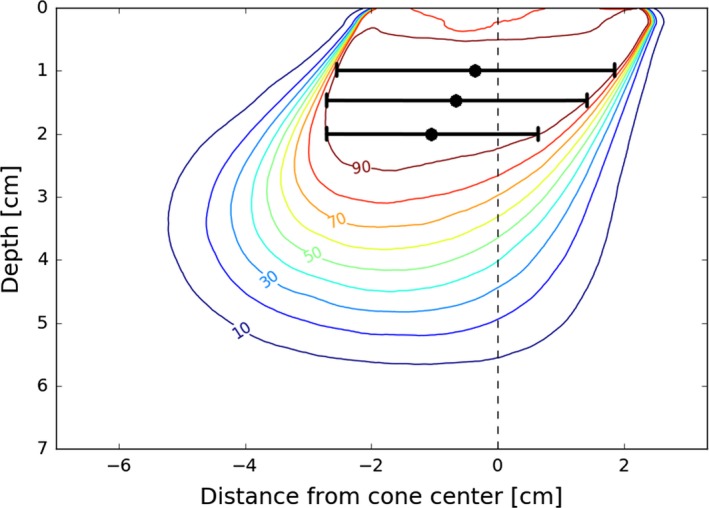
D_90_ tabulation scheme. The extent of d_90_ coverage and its central position was tabulated as a function of depth. The extent is represented for three depths as the length of the black bars, with the central position indicated by black circles. These were recorded in 1 mm increments at depth equal to or greater than d_max_.

The extent of d_90_ coverage in the in‐plane direction and the central position of the coverage (for beveled applicators) relative to the applicator's center were tabulated for each measured 2D profile. This is illustrated in Fig. 2. Depths greater than or equal to d_max_ were considered in 1 mm increments. To generate values for intermediate applicators, the d_90_ extent was linearly interpolated at each depth. The position of d_90_ coverage was modeled as a linear function of depth for each combination of energy and bevel angle.

**Figure 3:**
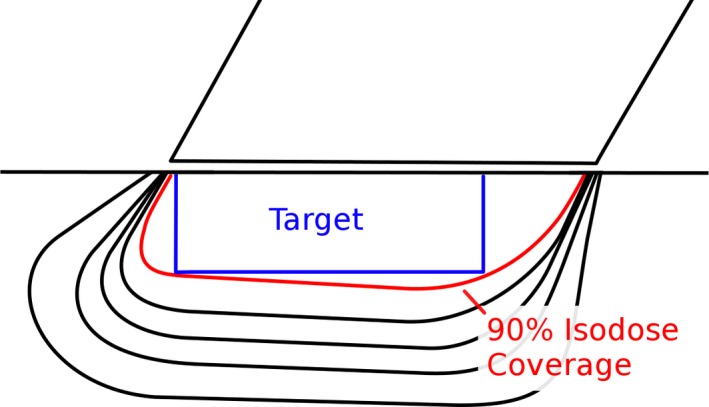
Applicator alignment example. The lookup tables assume that the front of the (beveled) applicator is just beyond the border of the target. This takes advantage of the characteristic shape of the 90% isodose coverage: the ‘leading edge’ of the coverage (i.e., the left hand side of the red line in the figure) exhibits a consistent lateral position with depth, whereas the ‘trailing edge’ varies considerably in its lateral position. The former conforms well to the hypothetical target, whereas the latter does not.

The tabulated d_90_ coverage was then used to generate a simple lookup table specifying an appropriate cone size and energy for a given bevel angle and target dimension. The target is assumed to be a cylinder with a user specified diameter and depth. This was accomplished with a simple algorithm: for each energy, each applicator is considered in ascending order of diameter. The tabulated extent and location of d_90_ coverage for the applicator is compared against the location of the hypothetical target as a function of depth. It is assumed that the front of beveled cones are aligned with the border of the target to maximize the use of d_90_ coverage, as illustrated in Fig. 3. The smallest cone that achieves coverage at all depths (if there are any) is determined for each energy. Finally, the energy that minimizes the volume of irradiated tissue is selected. The product of the depth of d_90_ and the cone diameter was used as an approximate surrogate for the volume of irradiated tissue.

Coverage in the cross‐plane dimension was based on transverse profiles measured with film for selected bevel and energy combinations. The minimum ratio of cross‐plane d_90_ coverage to in‐plane d_90_ coverage for each bevel‐energy combination was determined. The tabulated d_90_ in‐plane coverage was multiplied by this ratio to conservatively estimate cross‐plane coverage.

To validate film measurements, PDDs from film were compared to PDDs measured with the electron diode and with a parallel plate chamber. In‐plane profiles were also compared between film and the electron diode measurements.

### 2.D IORT procedures

Integrating the IORT program into the surgical procedures was accomplished through a series of coordinated meetings between staff members in the Department of Radiation Oncology and several different surgical specialties. This included physicians, physicists, radiation therapists, OR nurses, OR managers, and administrators.

Clinical use of the Mobetron was accompanied by QA measurements before the patient was brought into the room. These measurements were accomplished using a NE2505/3A farmer chamber (Nuclear Enterprises Ltd., Fairfield, NJ, USA) connected to an electrometer (Keithley Instruments, Cleveland OH, USA) and inserted into the manufacturer‐provided phantom. Charge per monitor unit (typically 200 MU's were used) were measured for all three energies at the depth of d_max_ and the nominal depth of d_50_. The d_max_ reading served to check the output, and the d_50_ reading divided by the d_max_ reading was used to check the energy. These were compared to commissioning benchmarks and tracked over time. Trends in the output and energy ratio were determined by performing least‐squares regression on the measurements over time. Statistical significance was determined by computing a two‐sided p‐value for the hypothesis that the slope of the fitted line was zero, with Bonferroni correction for multiple tests.

## 3 Results

### 3.A Radiation protection

Measurements of the instantaneous dose rate for the three energies were performed at 1 foot from all walls of the OR (one OR, two hallways, and the adjacent room housing the Mobetron control unit). It was also measured below the OR (a service driveway). Measured dose rates ranges from approximately 9 μSv per hour in the control area up to 90 μSv per hour in an adjacent hallway. The latter corresponds to 0.15 μSv per 1 Gy of delivered dose. Given the measured exposure rates, six or fewer patients per week can be treated in order to stay below regulatory limits (assuming an average patient dose of 20 Gy). This is about twice the permitted workload that would be determined using the table provided by Krechetov et al.,^18^ reflecting that the new design of the Mobetron was intended to reduce leakage radiation. In practice, two patients per week are the most that have been treated. QA measurements need to be scheduled with this in mind and occupancy in adjacent rooms may need to be controlled.

### 3.B Dosimetric measurements

#### 3.B.1 Tg‐51

Absolute calibration using the TG‐51 protocol was performed in the same manner as with isocentric linacs using a calibrated ion chamber and electrometer. Accuracy of the calibration was confirmed by means of the Radiological Physics Center (currently IROC‐Houston) service.

#### 3.B.2 Output factors and airgap measurements

**Figure 4:**
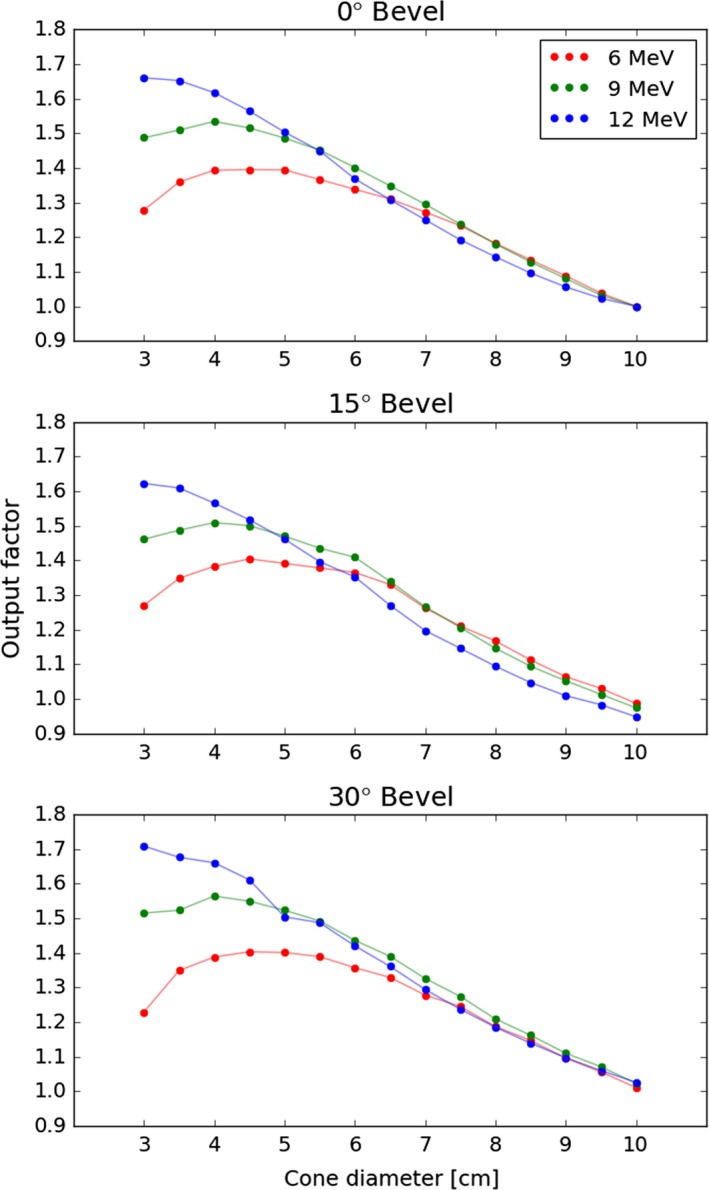
Output factors for each energy, bevel, and applicator diameter combination. Output factors are normalized to the measurement for the 0° bevel, 10 cm diameter applicator for each energy.

The measured output factors for the Mobetron have the unusual property of increasing with smaller applicator sizes. Output factors for each energy‐bevel combination are plotted in Fig. 4. The maximum output factors are at 5, 4, and 3 cm diameters for 6, 9, and 12 MeV, respectively. The maximum values are approximately 1.4, 1.5, and 1.7. Only a few output factors (30 degree bevel, 9.5 and 10 cm diameter) were less than 1. This represents a marked departure from the previous Mobetron model, where the maximum output factor was approximately 1.2 and some output factors were significantly less than 1.^15^

**Figure 5:**
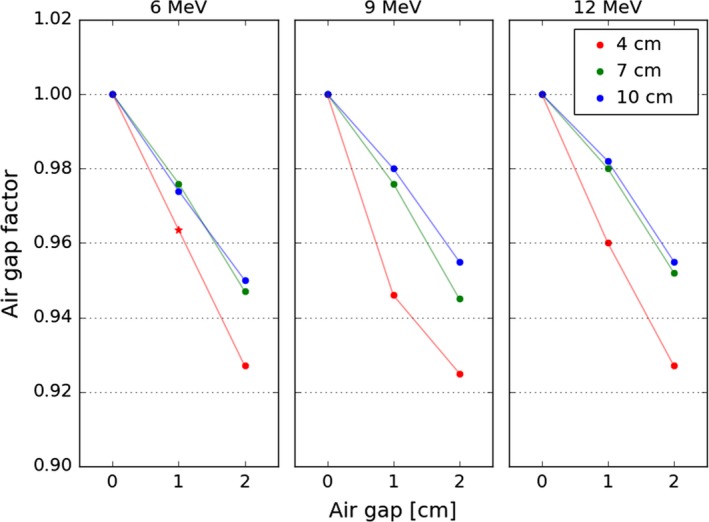
Air gap factors for each energy for 4, 7, and 10 cm diameter applicators. Diameter is the primary determinant of the air gap factor, changing energy has little effect. Larger applicators exhibit similar factors, with noticeable differences only manifesting for the 4 cm diameter cone. An interpolated value for 6 MeV is indicated by a star.

Air gap factors were quite similar across energies. The 7 and 10 cm cone air gap factors were similar, approximately 0.98 and 0.95 at 1 and 2 cm, respectively. The air gap factors for the 4‐cm cone were generally 2% lower than the larger cone sizes. Air gap factors are plotted in Fig. 5.

#### 3.B.3 Percent depth dose and lateral profiles

The surface dose, depth of d_max_, R_90_, R_80_, and Rp are listed for each energy in Table 1. These were determined from PDDs acquired with the 10 cm, 0° bevel applicator in place. E_p,0_ and Ē_0_ are also provided, derived from the PDDs in accordance with TG‐25.^23^ The depth of d_max_, R_90_, and R_80_ values are comparable to those published by Mills et al. for the Mobetron 1000, falling largely within 2 mm of the values reported there.^15^ The 12 MeV beam exhibits larger differences, up to 5 mm for R_80_.

**Table 1:** Electron beam parameters, 10 cm applicator and 0° bevel

Parameter	Nominal energy		
	6 MeV	9 MeV	12 MeV
Surface dose	86%	90%	94%
d_max_	1.15 cm	1.85 cm	1.93 cm
R_90_	1.73 cm	2.81 cm	3.88 cm
R_80_	1.96 cm	3.13 cm	4.37 cm
R_p_	3.08 cm	4.74 cm	6.40 cm
E_p,0_	6.34 MeV	9.66 MeV	12.99 MeV
Ē_0_	5.61 MeV	8.81 MeV	12.13 MeV

Generally speaking, using smaller diameter applicators had little effect on the measured PDD. Smaller applicators did moderately reduce the surface dose: changing from a 10‐cm cone to a 4‐cm cone reduced surface dose by 5% on average. For 12 MeV, the 4‐cm cone exhibited a slightly reduced range in water: R_90_ and R_50_ were approximately 4 and 3 mm shallower, respectively, than for the other larger cones.

**Figure 6:**
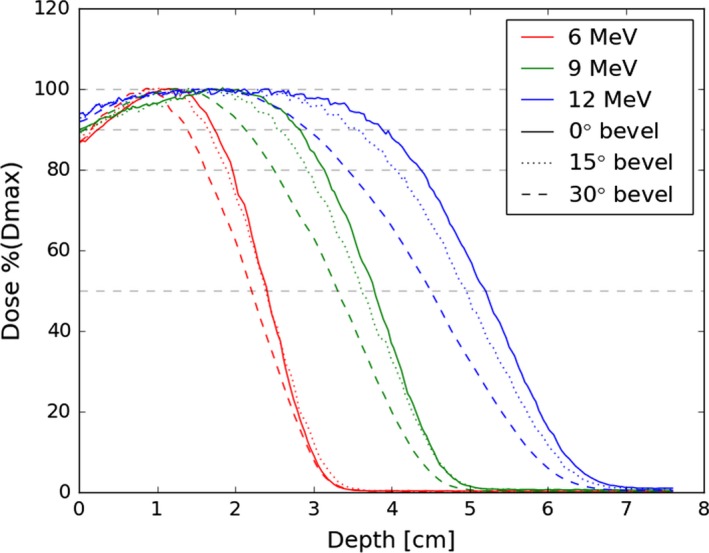
PDDs for the 0, 15 degree, and 30° 10‐cm applicators. Dashed lines indicate 100%, 90%, 80% and 50% of d_max_.

Changing the bevel angle had a more pronounced effect on the PDD, as expected given that the measurement axis did not coincide with the applicator axis. The 0° and 15° bevel PDDs are similar, within approximately 2 mm of one another. The 30° bevel introduces a more noticeable change. Selected PDDs are plotted in Fig. 6.

**Figure 7:**
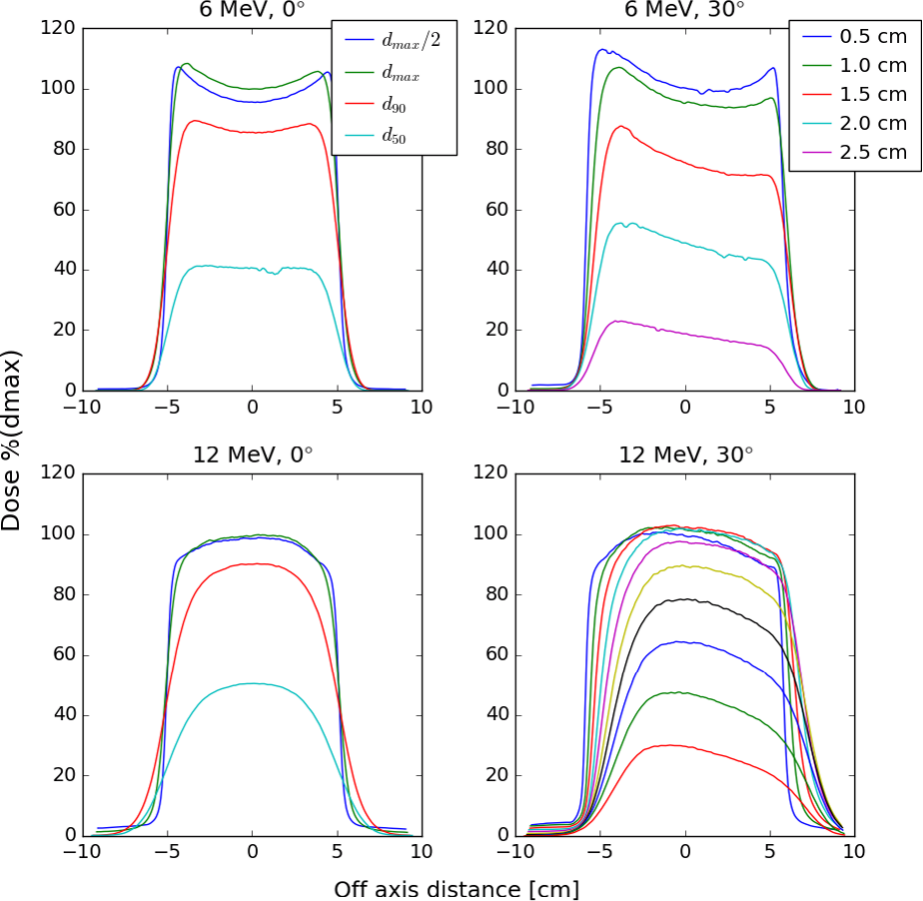
Lateral profiles measured with an electron diode in a scanning water tank. Profiles for 6 MeV (top) and 12 MeV (bottom) are displayed for both 0° bevel (left) and 30° bevel (right) applicators. All applicators are 10 cm in diameter. 0° bevel profiles are at depths of d_max_/2, d_max_, d_90_, and d_50_. 30° bevel profiles are at depths of integer multiples of 0.5 cm. The 6 MeV profiles exhibit noticeably sharper falloff than the 12 MeV profiles. The beveled profiles maintain some symmetry but are displaced from central axis.

Lateral profiles exhibited an increased dose toward the field edge for 6 MeV beams, and reduced dose toward the edge of the field for 12 MeV beams. In‐plane profiles for beveled applicators maintained some symmetry with an offset in the central position. Selected profiles are plotted in Fig. 7.

#### 3.B.4 2D Dose distributions

**Figure 8:**
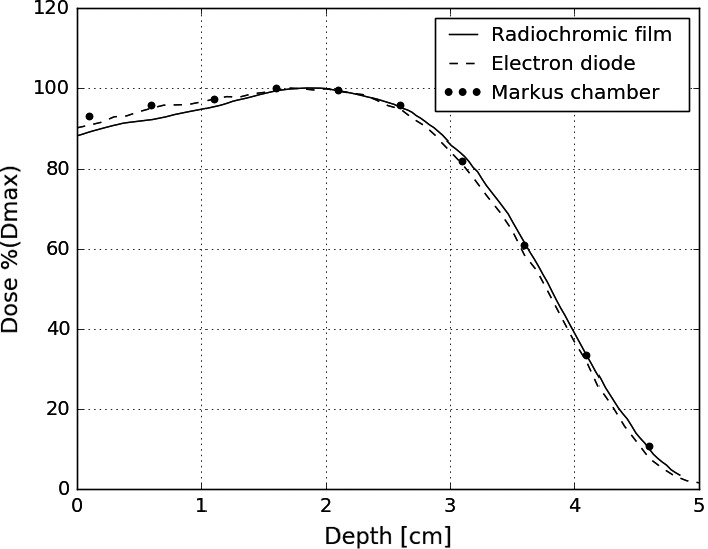
PDDs for a 9 MeV beam measured with radiochromic film, an electron diode and a plane‐parallel ion chamber.

**Figure 9:**
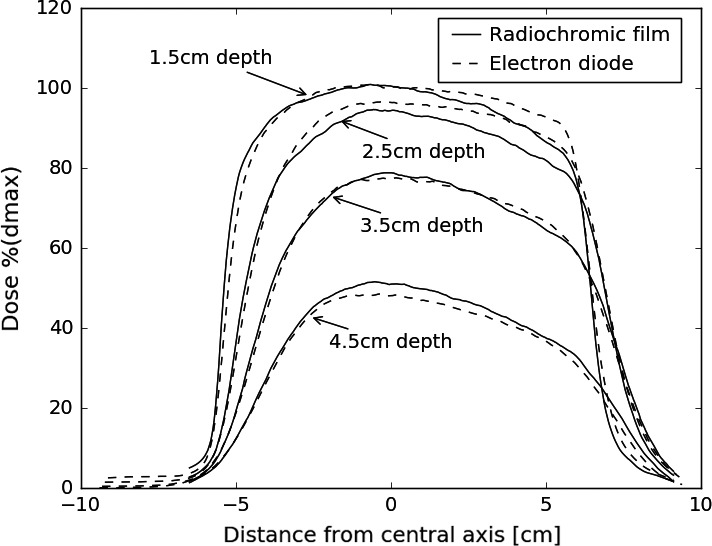
Comparison of in‐plane profiles measured with radiochromic film and an electron diode for a 12 MeV beam using a 10 cm diameter, 0° bevel applicator. The film under‐responds in one shoulder of the 1.5 and 2.5 cm depth profiles. However, this is not systematic, other film‐diode comparisons exhibited the opposite. More likely, it reflects the inherent uncertainties associated with film dosimetry.

Profiles from film measurements were spot‐checked against diode scans and ion chamber measurements (for PDDs) for validation purposes. Film was found to accurately reproduce these profiles, though artifacts were observed at shallow depths for some film profiles. Comparison of film, diode, and ion chamber profiles are displayed in Figs. 8 and 9.

**Figure 10:**
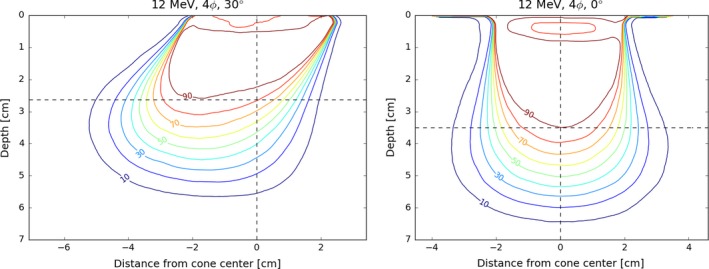
Comparison of the in‐plane profiles for a 12 MeV, 4 cm diameter applicator. Dashed lines indicate the maximum depth of D_90_ coverage and the center of the beam at the entrance. Using a 30° bevel reduces the depth of D_90_ by almost 1 cm in this case. The D_90_ coverage is also noticeably offset from the center of the applicator.

**Figure 11:**
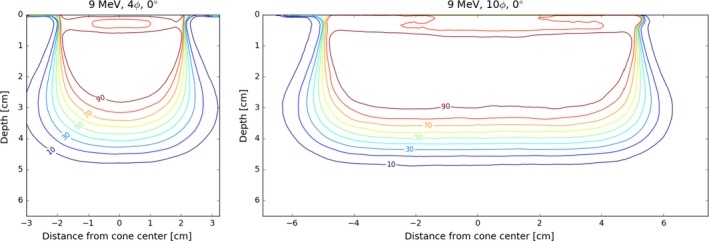
Comparison of in‐plane profiles for a 9 MeV beam with different diameter applicators. Applicator diameter has little effect on PDD characteristics.

**Figure 12:**
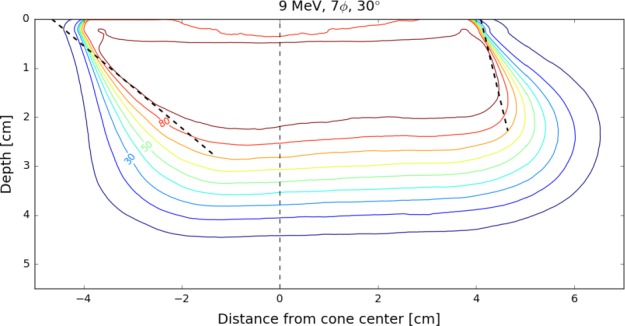
Example of the effect of applicator bevel for a larger diameter cone. D_90_ coverage is noticeably offset from the center of the applicator. The leading edge of D_90_ coverage exhibits minimal change in its lateral position as a function of depth, while the trailing edge exhibits a large change. This is a shared characteristic of distributions for beveled applicators.

Selected isodose distributions are presented in Figs. 10, 11, 12, illustrating general features of the in‐plane dose distributions as a function of bevel angle and diameter. Isodose constriction is clearly observed.

Lookup tables of applicator diameter energy combinations for 90% isodose coverage are presented in Tables 2, 3, 4. For superficial targets, a cone diameter slightly larger than the target (e.g., 0.5–1 cm) was sufficient. For targets with significant depth extent, the required cone size increased in order to offset isodose constriction. This was exacerbated for targets requiring beveled applicators, as the applicator size must account for both isodose constriction and the fact that the cone axis is not normal to the surface.

**Table 2:** Energy/cone diameter [MeV/cm] lookup for 0° bevel applicators

Target depth [cm]	Target diameter [cm]									
	1	2	3	4	5	6	7	8	9	9.5
0.4	6/3	6/3	6/4	6/5	6/6	6/7	6/7.5	6/8.5	6/9.5	6/10
0.8	6/3	6/3	6/4	6/5	6/6	6/7	6/7.5	6/8.5	6/9.5	6/10
1.2	6/3	6/3	6/4	6/5	6/6	6/7	6/7.5	6/8.5	6/9.5	6/10
1.6	6/3	6/3.5	6/4.5	6/5.5	6/6	6/7	6/8	6/9	6/10	‐
2.0	6/4	9/3.5	9/4	9/5	9/6	9/7	6/9	6/10	‐	‐
2.4	9/3	9/4	9/4.5	9/5.5	9/6.5	9/7.5	9/8.5	9/9.5	‐	‐
2.8	12/3	12/3.5	12/4.5	12/5.5	12/6.5	12/7.5	9/9.5	‐	‐	‐
3.2	12/3.5	12/4.5	12/5.5	12/6	12/7	12/8.5	‐	‐	‐	‐
3.6	12/5	12/6.5	12/7	12/7.5	12/10	‐	‐	‐	‐	‐
4.0	‐	‐	‐	‐	‐	‐	‐	‐	‐	‐

**Table 3:** Energy/cone diameter [MeV/cm] lookup for 15° bevel applicators

Target depth [cm]	Target diameter [cm]									
	1	2	3	4	5	6	7	8	9	9.5
0.4	6/3	6/3	6/4	6/5	6/6	6/7	6/8	6/9	6/10	‐
0.8	6/3	6/3	6/4	6/5	6/6	6/7	6/8	6/9	6/10	‐
1.2	6/3	6/3.5	6/4.5	6/5.5	6/6.5	6/7.5	6/8.5	6/9	6/10	‐
1.6	9/3	9/3.5	9/4.5	9/5.5	9/6.5	9/7.5	9/8.5	6/9.5	‐	‐
2.0	9/3	12/3.5	9/5	9/6	9/6.5	9/7.5	9/9	9/10	‐	‐
2.4	12/3	12/4	9/5.5	9/6.5	9/7	9/8.5	9/9.5	‐	‐	‐
2.8	12/3.5	12/4.5	12/5.5	12/6.5	12/7	12/9.5	‐	‐	‐	‐
3.2	‐	‐	‐	‐	‐	‐	‐	‐	‐	‐

**Table 4:** Energy/cone diameter [MeV/cm] lookup table for 30° bevel applicators

Target depth [cm]	Target diameter [cm]									
	1	2	3	4	5	6	7	8	9	9.5
0.4	6/3	6/3	6/4	6/5	6/6	6/7	6/8	6/9	6/10	‐
0.8	6/3	6/3	6/4	6/5	6/6	6/7	6/8	6/9	6/10	‐
1.2	6/3	6/4	6/5	6/5.5	6/6.5	6/7.5	6/8.5	6/9.5	‐	‐
1.6	9/3	9/4	9/5	9/6	9/7	9/8	9/9	9/10	‐	‐
2.0	12/3.5	12/4.5	12/5.5	12/6.5	12/7.5	9/9.5	9/10	‐	‐	‐
2.4	12/4.5	12/5	12/6	12/7	12/9	‐	‐	‐	‐	‐
2.8	‐	‐	‐	‐	‐	‐	‐	‐	‐	‐

### 3.C IORT procedures

**Figure 13:**
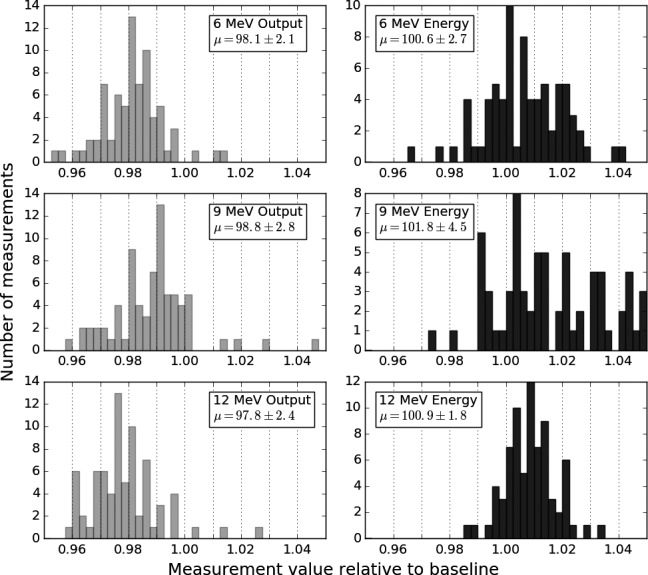
Daily QA measurements normalized to baseline measurements. The mean and 2 standard deviations are reported. Prior to each treatment a manufacturer supplied phantom was used to make measurements at each energy's nominal d_max_ and R_50_, as output and energy checks, respectively. Each histogram is the compilation of 76 such measurements since commissioning.

The results from 76 daily QA measurements (either pretreatment or as part of monthly QA) spanning 3 years are plotted in Fig. 13. The mean energy was found to be in good agreement with the baseline established at commissioning. The mean output was approximately 2% below the baseline for each energy. The observed daily variations are similar in magnitude to those reported by Beddar et al.^24^ for the Mobetron 1000, but larger than one would expect with a conventional linear accelerator. The outputs exhibited a statistically significant (all *P*‐values < 0.006) decreasing trend of approximately a 0.5% per year for all three electron energies. The energy ratio (d50/dmax) was stable for 6 and 9 Mev. The 12 MeV energy ratio exhibited an increasing trend of 0.3% per year that was just past the threshold of statistical significance (*P* = 0.049). Subsequent output measurements have fallen closer to the historic mean, thereby reducing the likelihood that the observed apparent decline in output was permanent or significant.

**Figure 14:**
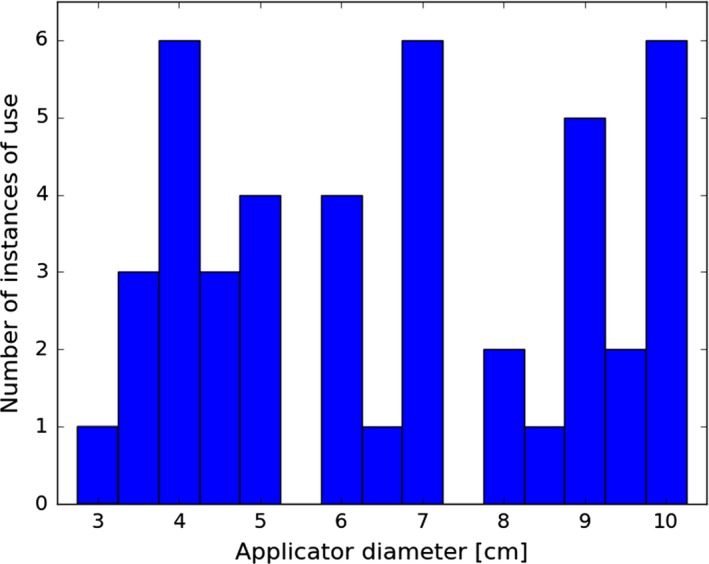
Usage statistics for different sizes of cones for the first 44 patient treatments.

Table 5 and Fig. 14 provide a detailed breakdown of the treatment parameters used for 44 patients treated at our institution. The majority of patients treated were sarcomas in the abdomen and pelvic regions. We have also treated several head and neck tumors.

**Table 5:** Treatment parameters for 44 patients

Parameter	Value	Number of patients
Energy	6 MeV	12
	9 MeV	32
	12 MeV	0
Bevel angle	0°	8
	15°	9
	30°	27
Bolus	0.0 cm	28
	0.5 cm	10
	1.0 cm	6

**Figure 15:**
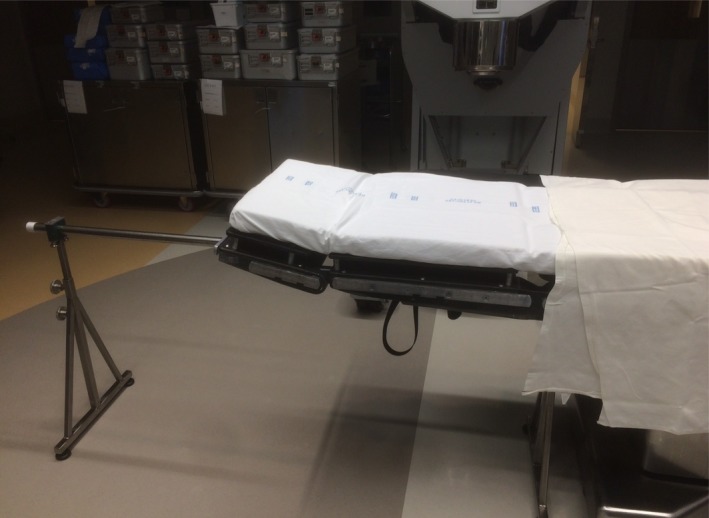
The in‐house bridge. For some resection sites, the surgical table must be extended far past the pedestal (visible in the bottom right hand portion of the photograph) to allow the Mobetron access, creating instability. The bridge — the metal rod suspended between two bases — provides stability and allows ample space for the legs of the Mobetron to come underneath without risk of collision.

One of the biggest issues during procedures was the type of operating table and its position relative to the Mobetron. The center of the Mobetron is approximately 56 cm from the outside edge of the leg of the Mobetron motorized lift. For an abdominal tumor, the patient must be positioned with his/her weight centered near the edge of the table creating problems of stability and patient support. A recommended table (Skytron LLC, Grand Rapids, MI, USA) has a greater capacity of extending the table top than most, but nevertheless, finding an achievable patient position, adequate table support, and Mobetron position was difficult in some cases due to potential collisions between the Mobetron legs and the moveable supporting legs for the table top. After about 2 years into the program, we developed an in‐house manufactured “bridge” to support the cantilevered table top to replace the moveable leg (Fig. 15). This support is being used routinely and has simplified and sped up the docking procedure markedly.

An important issue is the assignment of responsibilities for setting up the applicator and bolus sterilization and storage systems. If space is available, it is best to package, label, and sterilize each applicator separately. Boluses were packaged by cone size and bevel angle. Ease of access to the applicators is critical, but can be difficult to achieve while maintaining sterile procedures, especially since the Mobetron occupies considerable space.

Finally, it is critical for radiation oncology staff members to be familiar with OR procedures regarding sterility, privacy, and chains of responsibility. Equally important is the need for radiation protection awareness on the part of the OR personnel. We found that the different perspectives of the two cultures could be synergistic when there were good channels of communication and when each group was willing to adapt to the constraints and requirements of the other. Typical times for determining the appropriate treatment parameters, attaching the applicator to the table, moving the Mobetron into position, achieving proper alignment of the applicator, and irradiating takes approximately 45 minutes, although there is a wide range of times given the tumor location and the experience of the teams. This interval includes time for the surgeon and radiation oncologist to determine the exact region to treat, selecting and trying different applicators, moving the Mobetron into position, evacuation of the OR and adjacent areas, and the treatment itself.

## 4 Discussion

One of the advantages of the latest generation of intraoperative radiation sources is the ability to place them in existing operating suites. However, unless the OR is unusually isolated, there will be limitations on the number of monitor units that can be delivered.

An unusual feature of the Mobetron is the output factors, which increase with decreasing field size. The Mobetron is designed to achieve a flat profile with only one scattering foil for all energies and a fixed collimator, unlike conventional accelerators which have different foils and collimation settings for each energy. The Mobetron achieves a flat field through the design of the applicators. The applicators have no gaps, and electrons that would normally scatter out of the field will backscatter into the field, resulting in a flatter field. As the applicator size decreases, more electrons are backscattered to a smaller area, increasing the flux and accordingly the output factor. At some point, the applicator becomes small enough that the contribution to dose by backscattered electrons is offset by electrons lost due to the narrow bore (i.e., the entrance aperture), and the output factor begins to decrease. The difference in the output factors between the Mobetron 1000 and 2000 stems from differences in the applicator bores. The Mobetron 1000 bores were smaller to achieve a more consistent output factor. This made treatment field visualization more difficult, however, and increase lateral scatter from the machine as more electrons were intercepted by the bore. The Mobetron 2000 uses a wide bore design to rectify these issues, which results in relatively higher output factors.

During commissioning, we obtained the results of a survey of Mobetron 2000 output factors from four outside institutions. Comparisons of measured OF's with those of three other institutions provided support for our values. In general, all four institutions agreed within a few percent at large applicator sizes (greater than 6 cm) for all three energies. At smaller field sizes, there was considerably more divergence between measurements for all bevel angles, with the largest differences with the smallest applicator. For a 3‐cm applicator, our values were within 4% of the mean of the other institutions’ measurements for all energies and all angles. The differences between the other institutions’ measurements were of a similar magnitude.

Careful consideration should go into selecting the appropriate energy and applicator for treatment. If the required depth of irradiation is small, an applicator slightly larger than the target extent will be sufficient. As is typical of electron beams, skin sparing decreases with increasing electron energy. For superficial tumor volumes, it is sometimes advantageous to use a higher energy, e.g., 9 MeV rather than 6 MeV, and to add bolus to reduce the depth of penetration. In this way, adequate coverage of the most superficial cells is not compromised. However, if deep tissue needs to be irradiated isodose‐line constriction should ideally be accounted for to ensure the entire target volume receives sufficient dose. As treatment planning is currently done by hand in real time, this can be challenging. It is made more difficult if patient geometry requires the use of a beveled applicator that will skew the isodose distribution away from the axis normal to the tissue surface.

The applicator lookup table presented in this work is an attempt to address this challenge by consolidating the information from in‐plane dose distributions into a user‐friendly form amenable to real‐time use. It is intended to serve as a second check that parameters selected are reasonable. Examination of the different tables also serves as an illustrative example of the effects of isodose constriction and the effect of bevel angle. The former is illustrated by the increase in the applicator diameter as a function of depth in the 0° bevel table. The latter can be seen by comparing the maximum treatable depth between all three tables, or by comparing the required applicator diameter at a given depth between the tables. As an example of how the table might be used, consider a situation wherein the radiation oncologist would like to treat a 5 cm diameter area to a depth of 1.5 cm with 0.5 cm of bolus to ensure surface dose. Furthermore, suppose that given the geometry of the resection site either a 0° or 15° bevel may be appropriate. Using Table 2 for 0° bevel cones, one would look up the entry for a 5 cm diameter and 2 cm depth (the tables do not include bolus, so the bolus must be added to the depth to be treated), which is 9 MeV/6 cm cone. For the 15° bevel cone, Table 3 indicates that a 9 MeV/6.5 cm cone would be appropriate. One of these two can then be selected based on the geometry of the resection site in light of the required cone size.

The tables have clear limitations. First, they are based mostly on interpolated data between three sets of applicator sizes. However, measurements to spot check intermediate sizes indicated that this was a reasonably accurate assumption. They do not include margins to account for uncertainty, although they are based on conservative assumptions such as using the minimal ratio of cross‐plane to in‐plane coverage as a representative value. Nonetheless, they are not intended for use as a sole determinant of applicator and energy selection, but rather as a second check. Finally, the tables do not consider surface dose. Instead it is assumed that adequate surface dose will be achieved using bolus or selecting an appropriate energy. In the case bolus is used, the correct table target depth to use is the sum of the bolus thickness and depth of tissue to be treated.

We have not encountered a situation wherein the available applicators were not large enough to encompass the tumor bed. However, there are publications detailing field matching for exceptionally large treatment areas,^25^ typically using lead shielding to create a sharp, linear, well defined field border. For the Mobetron 1000, Beddar et al. found that a slight overlap for 4 and 6 MeV fields was necessary to prevent cold spots, and abutting fields were sufficient for higher energies.

Ease of clinical use of the Mobetron follows a steep learning curve. We have found that the positioning of the patient in order to achieve access for treatment has the largest effect on the time in the OR and successful irradiation. The center of the Mobetron is approximately 56 cm from the edge of the leg of the motorized jack, which means that the bed must be extended sufficiently. Abdominal and pelvic tumors can cause problems since the center of the patient's mass is cantilevered beyond the OR couch support. We have designed a “bridge” that provides solid support for the table but has sufficient width to allow access of the jack under the couch. Anticipating possible collisions is critical to reducing the time needed to position. The beamstopper can cause problems if the gantry angle needed is large. Occasionally, we have also found that the height of the OR or limitations on the vertical travel of the Mobetron can cause problems with certain patients and anatomical sites. Depending on the frequency of use, it is important to set up a regular schedule for running the Mobetron as we have found that there are fewer faults when it is used regularly (we have found monthly QA measurements to be adequate). In our practice, we routinely have a combination of three physicists and radiation therapists, in addition to the radiation oncologist, present during the procedure. This provides adequate manpower for positioning the Mobetron, keeping an eye out for potential collisions above and below the operating table, aligning the applicator and performing and checking the monitor unit calculations. This may reflect the wide diversity of tumor types and sites that we treat which reduces our ability to develop a more routine, streamlined process.

## 5 Conclusion

Intraoperative radiation therapy with the Mobetron has become a standard procedure that the Department of Radiation Oncology provides to the hospital surgical services. Commissioning is a fairly lengthy procedure given the large number of applicators and lack of dedicated access to a properly shielded room. Integration of radiation oncology staff members and the Mobetron into the operating room setting is a challenge with a steep learning curve, but a cooperative, proactive approach can greatly ameliorate these issues.
